# Identification and Modification of *Porphyromonas gingivalis* Cysteine Protease, Gingipain, Ideal for Screening Periodontitis

**DOI:** 10.3389/fimmu.2020.01017

**Published:** 2020-06-05

**Authors:** Kimito Hirai, Tomoko Yamaguchi-Tomikawa, Toru Eguchi, Hiroshi Maeda, Shogo Takashiba

**Affiliations:** ^1^Department of Pathophysiology—Periodontal Science, Graduate School of Medicine, Dentistry and Pharmaceutical Sciences, Okayama University, Okayama, Japan; ^2^R&D, Sunstar Inc., Osaka, Japan; ^3^Department of Endodontology, Osaka Dental University, Osaka, Japan

**Keywords:** screening chronic periodontitis, *Porphyromonas gingivalis*, serum IgG test, gingipain, specific antigen

## Abstract

Chronic periodontitis is an inflammatory disease caused by the formation of oral microbial biofilms. Periodontitis is associated with general health and not only oral diseases. *Porphyromonas gingivalis* is a well-known keystone pathogen for periodontitis and is associated with several systemic diseases, such as diabetes mellitus and Alzheimer's disease. We previously developed a system for screening periodontitis using *P. gingivalis*-specific serum immunoglobulin G (IgG) in an enzyme-linked immunosorbent assay with a sensitivity of 0.774 and a specificity of 0.586 and an area under the receiver operating characteristic curve of 0.708. However, the antigens elicited non-specific responses, since they were obtained from whole extracts of sonicated cultured bacteria. The purpose of this study was to identify antigens ideal for a sensitive and specific serum test. We identified the specific antigens using immunoaffinity columns immobilized with IgG antibodies from periodontitis patients. Liquid chromatography-tandem mass spectrometry identified 29 antigens from the elutes. Recombinant proteins for these candidates were synthesized using the wheat germ cell-free translation system and screened by dot blot analysis with serum from the columns. Three of the 16 candidates that reacted showed strongest affinities upon dot blot analysis; they included outer membrane protein 28, cysteine proteases, lysine gingipain Kgp, and arginine gingipain RgpA. Outer membrane protein 28 was not suitable for screening *P. gingivalis* infection because of its high false-negative rates. Kgp and RgpA were unstable antigens since they underwent self-digestion. They were made stable by substituting the active cysteine residues in Kgp and RgpA with alanine using site-directed mutagenesis. Using the modified antigens, we demonstrated that the patient serum IgG level against RgpA was the highest among all the antigens expressed in *P. gingivalis*. Moreover, the N-terminus of recombinant RgpA was excellent in differentiating between diseased and non-diseased states (with sensitivity of 0.85, specificity of 0.9, and area under the curve of 0.915). Although dot blot analysis was the only experiment used, the N-terminus of RgpA is an excellent antigen to immunologically test for *P. gingivalis* infection, especially for estimating the risks for periodontitis-associated systemic diseases. In conclusion, we have developed a *P. gingivalis* antigen for screening periodontitis.

## Introduction

Periodontitis is an inflammatory immune response elicited by the polymicrobial infection of anaerobic Gram-negative bacteria in subgingival plaques (1). This inflammatory condition damages periodontal tissues and characteristically results in the loss of alveolar bone surrounding the teeth. Periodontitis includes localized as well as chronic weak systemic inflammation in the oral cavity (2). Recent studies have shown that periodontitis is associated with systemic inflammatory diseases. Severe periodontal inflammation deteriorates the glycemic control in patients with diabetes mellitus; moreover, periodontitis is a risk factor for atherosclerotic cardiovascular disease, non-alcoholic fatty liver disease, and Alzheimer's disease (3–8). Therefore, the level of periodontal inflammation and immune responses could be used to diagnose the risk factors for developing systemic diseases.

Periodontitis can be diagnosed upon general examination, such as the prevalence of periodontal pockets and extent of alveolar bone loss, that primarily show the degree of periodontal tissue damage. Humoral immunological responses against periodontal pathogens can be determined by measuring the serum immunoglobulin G (IgG) antibody level against periodontal pathogens using enzyme-linked immunosorbent assays (ELISA) (9). It is recognized that infection with periodontal bacteria leads to humoral immunological responses and elevates the serum IgG antibody levels against pathogens (9). *Porphyromonas gingivalis*, a black-pigmented Gram-negative anaerobic bacterium, has been considered to be the keystone pathogen in periodontitis (10). Thus, the progression of periodontitis is associated with high serum IgG antibody levels against *P. gingivalis* (8, 11–13). It has also been reported that the serum IgG antibody level against *P. gingivalis* reduces with decreasing bacterial counts in the periodontal pockets upon treatment (11–13). In our previous study, we have demonstrated the utility of serum IgG antibody level against *P. gingivalis* in screening chronic periodontitis patients (9, 14). A recent study showed that serum IgG antibodies against periodontal pathogens serve as potential biomarkers that can be used to determine the risk factors for systemic diseases (15, 16). Univariable logistic regression analyses have shown serum IgG levels against *P. gingivalis* to be associated with cardiovascular disease (15).

However, testing the serum IgG level for periodontal pathogens is not performed during a clinical examination. One of the reasons for this is that using antigens from sonicated preparations of cultured pathogens in the media results in non-specific immunoreactions by IgG antibodies. Another reason could be the difficulty in standardizing the bacterial antigens in ELISA. It is difficult to precisely control the components of sonicated preparations from one study to another. A variety of *P. gingivalis* antigens with high immunogenicity have been reported; thus, it is possible to utilize and standardize a single or a combination of purified recombinant antigens, thereby enabling the development of a more specific IgG level test for periodontitis. Comparisons of antigens eliciting high serum IgG levels against *P. gingivalis* as a whole need to be performed; antigen(s) that have the capacity to discriminate between periodontitis patients and healthy individuals and that can be used for diagnosis remain to be discovered.

The aim of this study is to identify the *P. gingivalis* antigen(s) that are specifically recognized by serum IgG with high levels in periodontitis patients as compared to those in healthy individuals. We employed liquid chromatography-tandem mass spectrometry (LC-MS/MS) for screening the antigens recognized by patient serum IgG antibodies. Mass spectrometry is a unique and powerful analytical tool in that it can directly analyze any biological molecule susceptible to ionization. Recently, these proteomic analyzes by MS have been essential tools especially in periodontics (17, 18). We identified 29 different components in *P. gingivalis* and demonstrated that RgpA was the most appropriate antigen for screening periodontitis patients.

## Materials and Methods

### Study Design

Supplementary Figure 1 shows a schematic procedure for this study. This study comprised three screening steps to identify the antigens specifically recognized by serum IgG antibodies in periodontitis patients compared to those in healthy subjects, thereby establishing a novel antigen for the diagnosis of periodontitis. The first step included the purification of the sonicated crude protein extract from *P. gingivalis* strains FDC381 and SU63 using immunoaffinity columns immobilized with patient serum IgG antibodies with a high level against *P. gingivalis* FDC381 and SU63. The elutes were collected from the affinity columns and antigens were identified by liquid chromatography–tandem mass spectrometry (LC-MS/MS). Recombinant proteins were synthesized using the wheat germ cell-free translation system based on the 29 antigens that were identified by LC-MS/MS. In the second step, the immunogenicity of the 29 recombinant antigens were determined by dot blot analysis with sera used in the immunoaffinity columns. The candidate antigens that reacted with the serum IgG antibodies were selected and used in the third screening step. In this step, we determined the serum IgG level against the candidate antigens in 20 periodontitis patients and 10 healthy subjects using dot blot analysis. Finally, receiver operative characteristic (ROC) curves were analyzed to determine their capability to discriminate between the antigens showing significantly higher IgG levels in periodontitis patients than those in healthy subjects as per a published protocol (14).

### Serum Samples

Serum was collected from a total of 26 patients with periodontitis (11 males and 15 females with a mean ± standard deviation age of 52 ± 12 years). The control group comprised 10 healthy subjects without alveolar bone loss (five males and five females with a mean ± standard deviation age of 34 ± 5 years). The characteristics of all the individuals are shown in Table 1. We obtained six sera samples from patients (b1–b3 and c1–c3) with high IgG levels against *P. gingivalis* FDC381 and SU63. These were used in the immunoaffinity columns as part of the first and second steps for screening as described above. Levels of the serum IgG against *P. gingivalis* FDC381 and SU63 were quantified by ELISA as described in previously (9).

**Table 1:** Profile of the healthy control group and periodontitis patients.

	**Number in figure**	**IgG level (FDC381)**	**IgG level (SU63)**	**Number of present teeth**	**Bone loss (Schei value)**
**Healthy**	H1	−0.383	0.155	28	N.D.
	H2	−0.322	−0.239	28	N.D.
	H3	−0.557	−1.367	28	N.D.
	H4	−0.152	0.425	28	N.D.
	H5	−0.536	−1.307	28	N.D.
	H6	−0.562	−1.201	28	N.D.
	H7 (a1)	−0.634	−1.446	28	N.D.
	H8 (a2)	−0.647	−1.365	28	N.D.
	H9	−0.635	−1.321	28	N.D.
	H10 (a3)	−0.666	−1.445	28	N.D.
**Periodontitis**	b1	5.450	10.180	N.A.	N.A.
	b2	7.550	5.070	N.A.	N.A.
	b3	13.320	5.850	N.A.	N.A.
	c1	1.800	0.060	N.A.	N.A.
	c2	15.110	9.080	N.A.	N.A.
	c3	13.360	5.520	N.A.	N.A.
	P1	1.093	0.054	17	77.06
	P2	2.365	1.558	17	55.59
	P3	0.000	−0.813	10	55.50
	P4	1.211	−0.504	27	53.33
	P5	1.912	0.269	28	51.79
	P6	1.052	−0.202	24	47.50
	P7	2.415	0.755	22	44.77
	P8	37.587	11.373	18	43.06
	P9	0.040	−0.179	18	42.22
	P10	3.901	3.246	25	38.80
	P11	0.647	1.137	29	11.03
	P12	1.207	0.648	30	15.83
	P13	2.474	0.603	29	16.90
	P14	0.000	−0.521	27	17.04
	P15	8.073	1.324	25	19.80
	P16	2.270	0.814	16	20.00
	P17	−0.147	0.510	28	20.54
	P18	18.572	4.248	30	20.67
	P19	0.095	−0.378	28	20.89
	P20	1.657	−0.454	17	23.53

*Serum samples were obtained from 10 healthy subjects without bone loss (five male and five females; mean ± standard deviation age, 34 ± 5 years) and 26 patients with periodontitis (11 males and 15 females; mean ± standard deviation age, 52 ± 12 years). Serum IgG levels against P. gingivalis FDC381 and SU63 were measured by enzyme linked immunosorbent assay. The extent of bone loss was measured by the methods of Schei. Schei indices were classified as: <25% represents mild bone loss and >35% represents severe bone loss*.

*IgG levels of healthy subjects were usually lower than 1. In addition, healthy subjects had 28 teeth and no bone loss was detected (shown as N.D.). Meanwhile, numbers of teeth and bone loss of periodontitis patients used for affinity column (b1-b3 and c1-c3) were not available (shown as N.A.)*.

Samples from 10 patients with mild alveolar bone loss (P11–P20 bone loss; 18.6 ± 7.2%) and 10 patients with severe alveolar bone loss (P1–P10 bone loss; 51.0 ± 21.5%) were used for performing dot blot analysis as part of the third screening step. Alveolar bone loss in periodontitis patients was measured by the methods described by Schei (19) and expressed by the Schei index. Severity of bone resorption was classified according to the Schei indices: <25% represents mild bone loss; 25–35% represents moderate bone loss; and >35% represents severe bone loss. All patients signed an informed consent form for participation in the study and for the use of their biological samples. This study was approved by the Ethical Committee of Okayama University Graduate School of Medicine, Dentistry, and Pharmaceutical Sciences (approval no. 624).

### Immunoaffinity Chromatography

To select the antigens recognized by serum IgG in periodontitis patients, we used immunoaffinity columns based on the interaction between specific immobilized IgG antibodies and their target antigens (20). As previously reported (21), IgG antibodies in the serum from periodontitis patients exhibited two recognition patterns against the total proteins extracted from *P. gingivalis* that were observed as clear bands (b1–b3) and smears (c1–c3) on the Western blots (Supplementary Figure 2). Each serum type recognized different sizes of the antigens. Therefore, we used the two types of sera in the immunoaffinity columns. The sera obtained from three individuals in each group (healthy, clear bands, and smears) were mixed with the binding buffer (50 mM tartaric acid buffer with 3 M sodium chloride; pH 9.0). After washing protein G Sepharose beads (GE Healthcare Bio-Sciences, Rydalmaere, NSW, Australia) with the binding buffer, the sera mixed with the binding buffer were reacted with protein G Sepharose beads in Econo-pac columns (Bio-Rad Laboratories, Hercules, CA) on a rocking platform. After that binding process, the columns were washed with binding buffer and then crosslinking was performed to covalently attach antibodies to Protein G supports using the crosslinker BS3 (Pierce). Before the crosslinking, a 50 mM solution of BS3 was prepared, by dissolving 10 mg of BS3 in 6.8 mL of crosslinker buffer (0.2 M triethanolamine hydrochloride, pH 8.0). Blocking was performed with 0.2 M ethanolamine hydrochloride (pH 8.0). Each column was washed with elution buffer (0.1 M glycine hydrochloride, pH 2.8) and stored for further use in 50 mM Tris(hydroxymethyl)aminomethane hydrochloride (Tris-HCl, pH 7.5).

Whole protein extracts of *P. gingivalis* FDC381 and SU63 were purchased from the Institute of Immunology Co., LTD (Tokyo, Japan) and were dissolved in phosphate-buffered saline containing 0.03% sodium dodecyl sulfate (SDS), 0.005% Brij-35, and 0.2% CHAPS. *P. gingivalis* FDC381 and SU63 extracts (133 μg/1.5 mL) were injected into the immunoaffinity columns to purify the antigens recognized by serum IgG antibodies. The columns were washed three times with 20 mM Tris-HCl containing 0.005% Brij-35, 500 mM sodium chloride, and 0.1% CHAPS (pH 7.5). The antigens were eluted from each column using 0.05% trifluoroacetic acid, lyophilized, and stored for future use.

### Sodium Dodecyl Sulfate-Polyacrylamide Gel Electrophoresis (SDS-PAGE) and Western Blot Analysis

SDS-PAGE and Western blotting were performed as described previously (22). After SDS-PAGE, gels were either subjected to the Western blotting protocol or stained with 10% (v/v) acetic acid, 45% (v/v) methanol, and 0.1% (w/v) Coomassie brilliant blue R-250 for 1 h and subsequently de-stained with 10% (v/v) acetic acid and 30% (v/v) methanol overnight. For western blot analysis, SDS-PAGE gels were blotted on polyvinylidene fluoride membranes (EMD Millipore, Billerica, MA, USA) using the iBlot Dry Blotting System (Thermo Fisher Scientific, Inc., Waltham, MA, USA) as described previously (22). Serum samples from periodontitis patients were used at a final dilution of 1:2,500 in tris-buffered saline (TBS; 10 mM Tris-HCl buffer, pH 7.5, 0.9% sodium chloride) containing 3% (w/v) skimmed milk (M-TBS) with 0.1% Tween 20. Horseradish peroxidase-conjugated goat anti-human IgG secondary antibody (Chemicon International, Inc., Temecula, CA, USA) was used for detection at a dilution of 1:5,000 in M-TBS.

### Liquid Chromatography-Tandem Mass Spectrometry (LC-MS/MS)

Antigens eluted from the immunoaffinity columns were separated by SDS-PAGE. Protein bands were excised using a scalpel and samples were subjected to trypsin in-gel digestion using the robotic InvestigatorTM ProGest workstation (Digilab, Inc., Hopkinton, MA, USA) that automatically performed the washing, alkylation, enzyme activation, and thermal incubation cycles. Analysis of the digested extracts in 0.1% formic acid were performed after LC-MS/MS using the C12 Jupiter high performance liquid chromatography column and LTQ Orbitrap XL mass spectrometer (Thermo Fisher Scientific, Inc.). The flow rate was maintained at 300 nL/min. Mass spectra were recorded in the data-dependent mode from the six most abundant ions. Raw data from the mass spectrometer were used as input for the Mascot database (www.matrixscience.com) with a peptide tolerance of ±10 ppm and an MS/MS tolerance of ±0.5; fixed modifications—carbamidomethyl; variable modifications—oxidation, acetylation, pyroglutamination, and deamidation; and up to two missed cleavages by trypsin (23). Protein and peptide matches were filtered and validated using the Scaffold algorithm (www.proteomesoftware.com) (23). Protein identity was accepted at a >95% probability and contained at least two identified peptides (at ≥95%).

### Construction of Recombinant Antigens

Recombinant proteins of the 29 antigens identified by LC-MS/MS were prepared using the wheat germ cell-free translation system (CellFree Sciences, Ehime, Japan) as described previously (24). The complete target genes including a sequence of signal peptide were amplified by polymerase chain reaction. The primers were designed (Supplementary Table 1) to contain restriction sites and attB1/B2 sequences for the subsequent cloning protocol. The attB1 sequence (5′-GGGGACAAGTTTGTACAAAAAAGCAGGCT-3′) was added to the 5′ ends of each of the forward primers and the attB2 sequence (5′-GGGGACCACTTTGTACAAGAAAGCTGGGT-3′) was added to the 5′ ends of each of the reverse primers. The reaction was performed in a volume of 50 μL using Ex-Taq polymerase (Takara Bio, Inc., Shiga, Japan) under the conditions recommended by the manufacturer with 20 pmol of forward and reverse primers and 100 ng of the cDNA. The reaction conditions included 35 cycles of denaturation at 98°C for 60 s, annealing at 58°C for 30 s, and extension at 72°C for 2.5 min with a final extension at 72°C for 10 min. We employed the Gateway cloning protocol by Invitrogen (Thermo Fisher Scientific, Inc.) for subcloning the genes of interest into a set of destination vectors (25). This molecular biology-based technique efficiently transfers DNA fragments between plasmids using a proprietary set of recombination sequences like the attB1/B2 sites. The modified target gene sequences were ligated into the pDONR221 vector (Thermo Fisher Scientific, Inc.). Subsequently, these plasmids were transformed into standard competent *E. coli* DH5α cells (Takara Bio, Inc.). Plasmids were isolated from cultured *E. coli* DH5α and digested with the respective restriction enzyme (Supplementary Table 1). The digested gene fragments were cloned into the corresponding sites of the expression vector pEU-E01-GST-TEV (CellFree Sciences) in frame with the gene encoding glutathione S-transferase (GST) at the N-terminus of the target gene.

Synthesis and purification of the recombinant proteins were performed using the CellFree Sciences kit. Briefly, transcription and translation were performed using the CFS-TRI-1240G kit (CellFree Sciences) and the expression plasmids, as described above. *In vitro* transcription was performed at 37°C for 6 h in a reaction buffer containing 100 ng/μL of the plasmids, 1 unit/μL of SP6 RNA polymerase, 1 unit/μL of RNase inhibitor, 2.5 mM NTPs, and 1 × transcription buffer that was supplied with the kit. *In vitro* translation was performed using 5.5 mL of SUB-AMIX (proprietary buffer containing all the amino acids) with 250 μL of the transcription products, 1 μL of creatine kinase (20 mg/mL), and 250 μL of WEPRO1240G supplied with the kit for 16 h at 17°C. Glutathione Sepharose 4B gel (GE Healthcare Bio-Sciences) was used to purify the GST-tagged proteins. Samples from each purification step were analyzed by SDS-PAGE.

### Inactivation of Protease Activity Using Cysteine Residue Mutants

Two of the recombinant antigens that we identified, Lys-gingipain (Kgp) and Arg-gingipain (RgpA), are strong cysteine proteases that are capable of self-digestion and, thus, are unstable. To prevent this self-digestion, the cysteine residues C477 on Kgp and C471 on RgpA were substituted with alanine residues by site-directed mutagenesis using the PrimeSTAR® mutagenesis basal kit (Takara Bio, Inc.) according to the kit protocol. The following primers were designed to introduce the mutations (the mutated regions have been underlined): C477 (TGT) in Kgp (5′-ATTACAGCTCAATTCGATTA-3′/5′-AGCGCAGTTGCCAATAGCTA-3′); C488 (TGC) in Kgp (5′-CCTTCGGAGAGGTAATAACT-3′/5′-CAGGCTGTACATAATCGAAT-3′); C471 (TGT) in RgpA (5′-GTGAATGGCGATTTCCTATT-3′/5′-AGCAGCTACGTCGAAAATAA-3′); C482 (TGT) in RgpA (5′-CTTTCGCAGAAGCATTGATG-3′/5′-CAGGCATGCTGAATAGGAAA-3′). The desired mutations were confirmed by DNA sequencing and the mutants were expressed and purified by the methods described above.

### Dot Blot Analysis

Dot blots were used to analyze the reactivity of recombinant antigens to patient serum IgG. The recombinant antigens were diluted with TBS to a final concentration of 12.5 μg/mL. Aliquots of 4 μL of the diluted proteins were spotted on nitrocellulose membranes (Bio-Rad Laboratories, Hercules, CA). After the spots had dried, the membranes were blocked with M-TBS. The blocked membranes were then incubated with serum at a dilution of 1:1,000 in M-TBS for 12 h at room temperature. The membranes were washed with TBS containing 0.05% Tween 20 and the antibody bound to the membrane was detected with horseradish peroxidase-conjugated goat anti-human IgG secondary antibody (Chemicon International, Inc.). After colorimetric detection using naphthol, the intensities of the spots on the blots were quantified using the computerized densitometry ImageQuant LAS4000 Digital System (GE Healthcare Bio-Sciences) and analyzed using the ImageQuant TL software (GE Healthcare Bio-Sciences). The spots on the blots were scanned in the “array analysis” mode. Blank areas without signals of the recombinant proteins were used for subtracting background noise and normalization.

### Statistical Analysis

Statistical analysis was performed using Ekuseru-Toukei 2015 (Social Survey Research Information Co., Ltd., Tokyo Japan). The Mann-Whitney *U* test was used to compare the differences in serum IgG levels between the periodontitis patients and healthy individuals. The cut-off values for IgG levels were obtained from the ROC curves. Diagnostic efficacy was calculated and represented as sensitivity and specificity. The area under the ROC curve was used to evaluate the capacity to discriminate between the periodontitis patients and healthy individuals. The values for area under the ROC curve (AUC) were categorized as: 0.5–0.7 represents no to low discrimination; 0.7–0.8 represents moderate discrimination; 0.8–0.9 represents excellent discrimination; and >0.9 represents outstanding discrimination.

## Results

### Identification of *P. gingivalis* Antigens Recognized by Periodontitis Patient Sera

**Figure 1:**
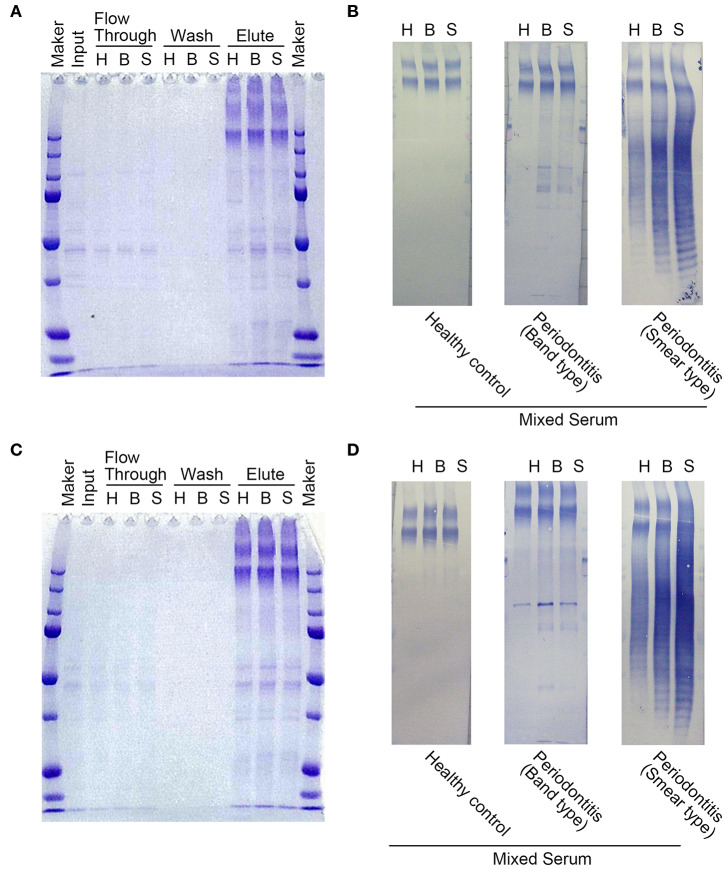
Protein contents in various fractions after purification using immunoaffinity columns. SDS-PAGE was used to separate the proteins from each fraction for strains FDC381 **(A)** and SU63 **(C)**. 10 μL of each fraction were loaded into each gel lane. Immunoreactivities of the separated proteins in the elutes for strains FDC381 **(B)** and SU63 **(D)** were confirmed by Western blot analysis with mixed serum used in the immunoaffinity columns as described in Supplementary Figure 2. Approximately, 2 μg of each protein was blotted on the membrane, and pooled sera used for affinity column (three subjects in each) were used at a dilution of 1:2,500. H: healthy control, B: the serum type of clear bands, S: the serum type of smear bands.

IgG antibodies in the serum of periodontitis patients exhibited two recognition patterns against the whole extracts of *P. gingivalis* including a group of clear bands or smears on Western blots (21). The clear bands (Supplementary Figure 2B) mainly corresponded to ~30–3 kDa antigens, while the smears (Supplementary Figure 2C) included a wide range of antigens (25–250 kDa). Moreover, it has been reported that the smears on the Western blots partly comprised the *P. gingivalis* lipopolysaccharide (21, 26). Therefore, we used these sera from two groups of periodontitis patients (b1-b3 and c1-c3 in Supplementary Figure 2) and that from healthy individuals (a1-a3 in Supplementary Figure 2) to purify the antigens using immunoaffinity columns. Whole protein extracts containing the *P. gingivalis* FDC381 and SU63 antigens that were recognized by the serum IgG antibodies were separated in the eluted fractions from the immunoaffinity columns (Figures 1A,C). As shown in Figures 1B,D, immunoreactivity of the separated proteins in the elutes was confirmed using mixed sera from the immunoaffinity columns (the clear bands, smears, and healthy individuals) by Western blot analysis. The purified antigens in the elutes of FDC381 and SU63 were analyzed by LC-MS/MS using the Thermo Fisher LTQ Orbitrap XL. As shown in Table 2, 29 antigens were identified by the Mascot database and Scaffold algorithm using LC-MS/MS data that were enriched in the patient sample columns as compared to the columns for the healthy group. Although we prepared immunoaffinity columns containing each group of serum IgG antibody on protein G Sepharose (clear bands and smears), the profile of the antigens eluted from each immunoaffinity column was mostly similar (data not shown). These antigens cosmprised six outer membrane proteins, six heme-binding proteins, nine enzymes, two fimbrillin proteins, and six unknown proteins. Many of the antigens have been reported to be strongly immunogenic, such as heme-binding proteins, outer membrane proteins, fimbrillins, and the gingipain family of enzymes. The antigens No.7, 11, 19, 26, and 29 were identified not only from patients' columns but also from healthy subjects' columns. However, spectral count obtained by LC-MS/MS defined as the total number of spectra identified for a protein from healthy columns was quite smaller than that from patient columns (Data not shown). Since the overlapped antigens had the possibility of showing higher antibody level in patient sera compared to healthy sera, we synthesized all the 29 antigens, identified and listed in Table 2.

**Table 2:** List of *P. gingivalis* antigens recognized by patients' serum IgG.

**No**.	**Identified proteins**	**Accession#**	**MW**	**Characteristics**
1	Hypothetical protein with Zinc carboxypeptidase domain	gi|188994199 (+1)	92 kDa	Unknown
2	Hypothetical protein PG1881	gi|34541489	53 kDa	Unknown
3	Hypothetical protein PGN_0291	gi|188994155	134 kDa	Unknown
4	Hypothetical protein PGN_1611	gi|188995475 (+1)	53 kDa	Unknown
5	Hypothetical protein PGN_0477	gi|188994341 (+1)	61 kDa	Unknown
6	Hypothetical protein PGN_0860	gi|188994724 (+1)	39 kDa	Unknown
7	53 kDa major outer membrane protein	gi|5832527	54 kDa	Outer membrane protein
8	35 kDa hemin binding protein	gi|188994523 (+1)	38 kDa	Iron binding protein
9	Iron hemin transport FetB	gi|188994569 (+2)	33 kDa	Iron binding protein
10	NAD-dependent glutamate dehydrogenase	gi|150842 (+1)	49 kDa	Enzyme
11	Phosphoserine aminotransferase	gi|34540980	40 kDa	Enzyme
12	TonB-linked receptor (Tlr)	gi|188994547 (+1)	79 kDa	Receptor
13	Fimbrilin	gi|34541709 (+1)	41 kDa	Fimbriae
14	Minor component FimE	gi|188994049	61 kDa	Fimbriae
15	HmuY	gi|119392294 (+1)	24 kDa	Iron binding protein
16	M24 family peptidase	gi|34540922	67 kDa	Proteinase
17	Glyceraldehyde 3-phosphate dehydrogenase, type I	gi|34541701	36 kDa	Enzyme
18	Ferritin	gi|34540987	19 kDa	Iron binding protein
19	Serine hydroxymethyltransferase	gi|34539916	47 kDa	Enzyme
20	Outer membrane lipoprotein Omp28	gi|34541744	32 kDa	Enzyme
21	Probable lysyl endopeptidase precursor	gi|188995280 (+1)	103 kDa	Proteinase
22	Quinone family NAD(P)H dehydrogenase	gi|34541436	20 kDa	Enzyme
23	DNA-binding protein from starved cells Dps	gi|188995900 (+2)	18 kDa	DNA binding protein
24	Immunoreactive 42 kDa antigen PG33	gi|34540489	42 kDa	Outer membrane protein
25	Lysine-specific cysteine proteinase (Lys-gingipain, Kgp)	gi|1314751	187 kDa	Proteinase
26	RagA protein	gi|34540042 (+1)	112 kDa	TonB-linked receptor antigen
27	Arginine-specific cysteine proteinase (Arg-gingipain, RgpA)	gi|188995833 (+2)	185 kDa	Proteinase
28	Outer membrane protein 41 precursor	gi|188994593 (+2)	43 kDa	Outer membrane
29	RagB lipoprotein	gi|34540043	56 kDa	Lipoprotein

*The antigens collected by immunoaffinity columns were analyzed using the Thermo Fisher LTQ Orbitrap XL (LC-MS/MS). The LC-MS/MS data were analyzed using the Mascot database (www.matrixscience.com) and Scaffold algorithm (www.proteomesoftware.com) for protein identification*.

### Selection of Recombinant *P. gingivalis* Antigens Recognized by Periodontitis Patient Sera

**Figure 2:**
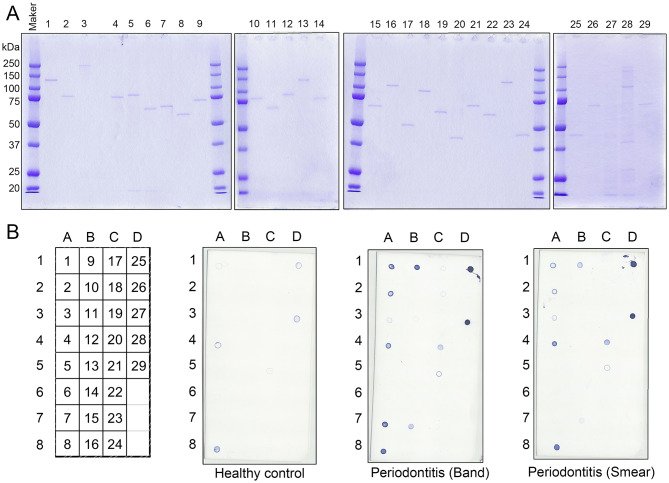
Immunogenicity of the recombinant *P. gingivalis* antigens synthesized using the wheat germ cell-free translation system (CellFree Science). **(A)** SDS-PAGE analysis of the recombinant proteins to confirm protein purity. The numbers exhibited on top of the SDS-PAGE gels were recombinant protein numbers shown in Table 2. Approximately, 50 ng of each recombinant protein was loaded into each lane. **(B)** Serum IgG responses to the recombinant proteins by dot blot analysis with mixed serum used in the immunoaffinity columns (clear bands, smears, and healthy individuals). Approximately, 50 ng of each protein was blotted on the membrane, and sera were used at a dilution of 1:2,500. The left panel shows a scheme of the proteins spotted on the membrane. The numbers exhibited on the left panel are the recombinant protein numbers shown in Table 2.

The 29 antigens identified and listed in Table 2 were translated *in vitro* using the wheat germ cell-free translation system. The purity of every recombinant protein was determined by SDS-PAGE (Figure 2A). Recombinant antigens No. 25, 27, and 28 were smaller than their predicted sizes or were observed as multiple bands. Due to their strong protease activity, recombinant antigen numbers 25 and 27 that were Lys-gingipain (Kgp) and Arg-gingipain (RgpA), respectively, underwent self-digestion and subsequently were unstable. As shown in Figure 2B, dot blot analyses confirmed the immunogenicity of each recombinant antigen against the mixed serum (comprising clear bands, smears, and healthy control samples) used in the immunoaffinity columns. Recombinant forms of the major outer membrane proteins RagA (antigen 26), RagB (antigen 29), and fimbriae proteins FimA (antigen 13) (27–29), that have been reported to be antigens inducing high levels of serum IgG, showed low or no signal on the blot (Figure 2B). The intensities of the spots for antigens No. 1–4, 7–9, 11, 15, 17, 18, 20, 21, 23, 25, and 27 were relatively strong using the periodontitis patient mixed serum as compared to those using serum from healthy individuals. These antigens included heme-binding proteins, the gingipain family of enzymes, and outer membrane proteins that have been reported to be antigens capable of inducing high levels of serum IgG or strong virulence factors. The remaining 13 recombinant antigens showed low or no signal on the blot.

### Inactivation of the Protease Activity of Antigens 25 and 27 (Gingipains) by Modifying Their Cysteine Residues

**Figure 3:**
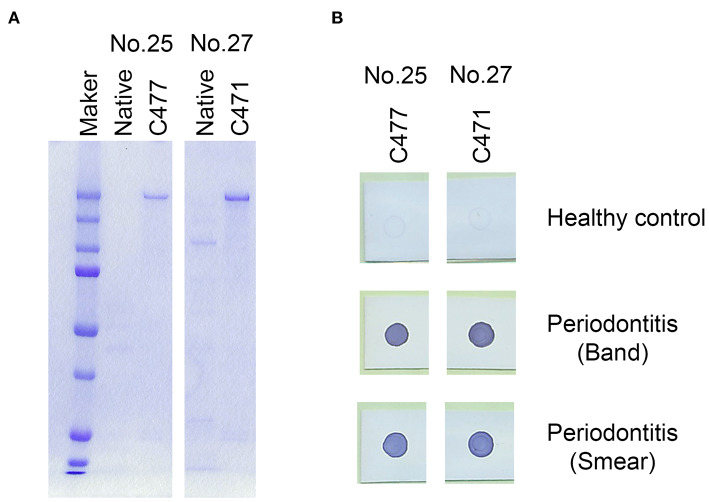
Modification of the cysteine proteases, Lys-gingipain (Kgp; antigen no. 25) and Arg-gingipain (RgpA; antigen no. 27) to inhibit their proteinase activities. Cysteine residues were substituted with alanine residues on C477 of recombinant Kgp and C471 on recombinant RgpA. **(A)** SDS-PAGE analysis of the mutant recombinant proteins. Approximately, 50 ng of each recombinant protein was loaded into each lane. **(B)** Serum IgG responses to the mutants as determined by the dot blot analysis. Approximately, 50 ng of each protein was blotted on the membrane, and sera were used at a dilution of 1:2,500.

Gingipains, a group of lysine (Kgp) or arginine (RgpA) specific cysteine proteases, are a major group of virulence factors in *P. gingivalis*. Due to their strong protease activity, we inactivated the protease activities of recombinant antigen numbers 25 (Kgp) and 27 (RgpA) and stabilized them. The protease activity of RgpA and Kgp are regulated by cysteine residues C471 on RgpA and C477 on Kgp, respectively (30–32). Subsequently, cysteine residues C477 in the recombinant Kgp and C471 in the recombinant RgpA were substituted with alanine residues to inhibit their protease activities. All the modified antigens maintained their immunogenicity against the serum obtained from the periodontitis patients (Figure 3B). Therefore, we used the 25A and 27A antigens wherein C477 and C471 were substituted with alanine residues, respectively, for further experiments.

### Serum IgG Level Against Candidate Antigens Obtained by Dot Blot Analysis

**Figure 4:**
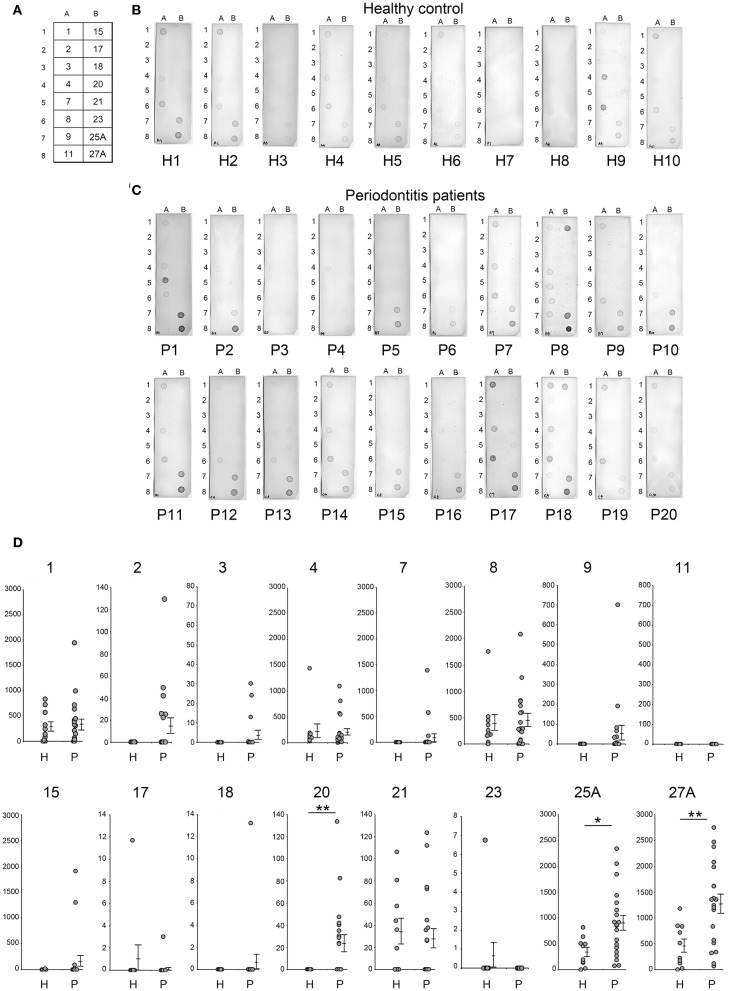
Analysis of serum IgG responses to recombinant antigens. **(A)** The panel shows a schematic of the proteins spotted on the membrane used for dot blotting. The numbers exhibited on the panel were recombinant protein numbers shown in Table 2. The results of individual serum from healthy controls (**B**: H1–H10) and patients with periodontitis (**C**: P1–P20) have been shown. The 20 periodontitis patients that comprised this experiment included 10 patients each with mild alveolar bone loss (18.6 ± 7.2) or severe alveolar bone loss (51.0 ± 21.5) as measured by the method of Schei. Approximately 50 ng of each protein was spotted on the membrane and sera were used at a dilution of 1:2,500. Spot intensities were used for analysis of the dot blots. **(D)** Spot intensities were normalized relative to the background signals, and represented as relative intensities. Mean values and standard errors are shown on the right. **P < 0.01; *P < 0.05; H, Healthy control; P, Patients with periodontitis.

In this study, serum IgG levels were quantified using the intensities of the spots from the dot blots. Dot blot analysis included 20 patients with periodontitis and 10 healthy individuals. Periodontitis patients comprised 10 patients each with mild alveolar bone loss (P11-P20) or severe alveolar bone loss (P1-P10) as measured by the methods of Schei (19). Antigens No. 1–4, 7-9, 11, 15, 17, 18, 20, 21, 23, 25A, and 27A were selected from our previous results (Figures 2, 3) and spotted on the membrane (Figure 4A), that was then incubated with the serum (Figures 4B,C). As shown in Figure 4D, the intensities of the spots on each membrane were quantified using ImageQuant TL as described in Materials and Methods. Although the average IgG level against antigens No. 1, 4, 8, and 21 was relatively high, the interaction was not specific to the serum from periodontitis patients. The levels for the periodontitis patient's serum IgG against antigens number 20 (*p* = 0.009), 25A (*p* = 0.013), and 27A (*p* =0.005) were significantly higher than those using the serum from healthy individuals. Serum IgG levels did not correlate with the severity of alveolar bone loss (data not shown).

### ROC Curve Analysis for the Potential Use of Recombinant Antigens in the Diagnosis of Periodontitis

**Figure 5:**
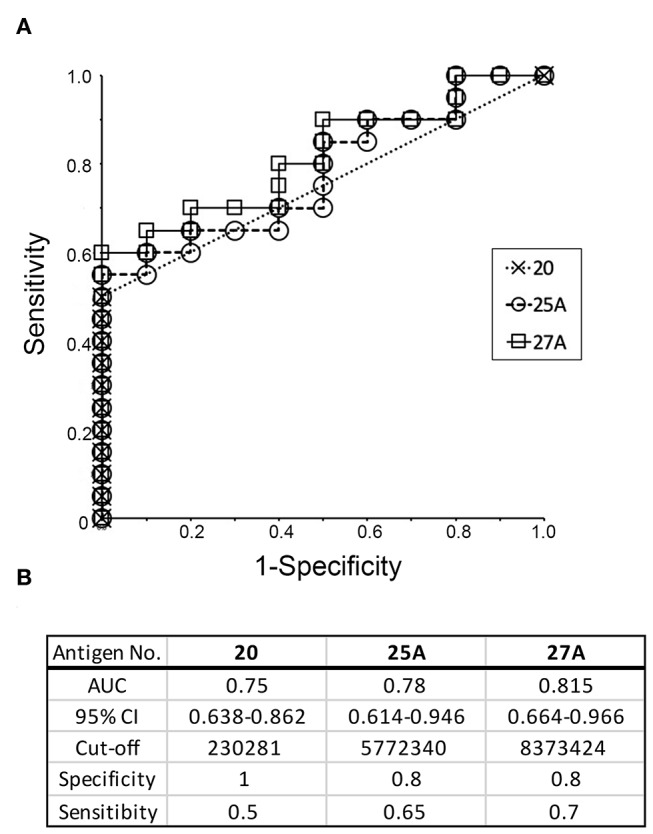
Receiver operative characteristic (ROC) curve analysis to evaluate the diagnostic capacity of individual recombinant antigens (20, 25A, and 27A) to distinguish between periodontitis patients and healthy individuals. **(A)** ROC curves were generated as described by Ekuseru-Toukei 2010 (Social Survey Research Information Co., Ltd.) using the spot intensities from the dot blot analysis (Figure 4). **(B)** The values of AUC with 95% confidence interval (CI) and sensitivity/specificity with optimal cut-offs are presented in the lower panel.

The ability of the recombinant antigens to distinguish periodontitis patients from healthy individuals was analyzed using ROC curves and the values for AUC. ROC curves for antigen numbers 20, 25A, and 27A were obtained by plotting the sensitivity and specificity values calculated using the spot intensities shown in Figure 4. The values for AUC with a 95% confidence interval and sensitivity/specificity with optimal cut-off have been presented in the lower panel of Figure 5. The values for the remaining recombinant antigens (1–4, 7–9, 11, 15, 17, 18, 20, 21, 23, 25A, and 27A) have been listed in Supplementary Figure 3. The values for AUC were categorized as: 0.5–0.7 that represents low to nil discrimination; 0.7–0.8 that represents moderate discrimination; 0.8–0.9 that represents excellent discrimination; and >0.9 that represents outstanding discrimination between periodontitis patients and healthy individuals. The values of AUC for antigens number 20 (outer membrane protein 28, Omp28: 0.75), 25A (Kgp: 0.78), and 27A (RgpA: 0.815) were higher than 0.7, suggesting a capacity to moderately differentiate periodontitis patients from healthy individuals. Although the AUC of Omp28 signified moderate discrimination of the samples (0.7–0.8), half of the patient sera (10 out of 20) did not completely recognize Omp28 (Figure 4D); thus, indicating a high false negative rate for this antigen. Therefore, we focused on Kgp and RgpA in our subsequent experiments.

### Using Mutant Antigens to Increase the Potential for Discrimination by RgpA and Kgp

**Figure 6:**
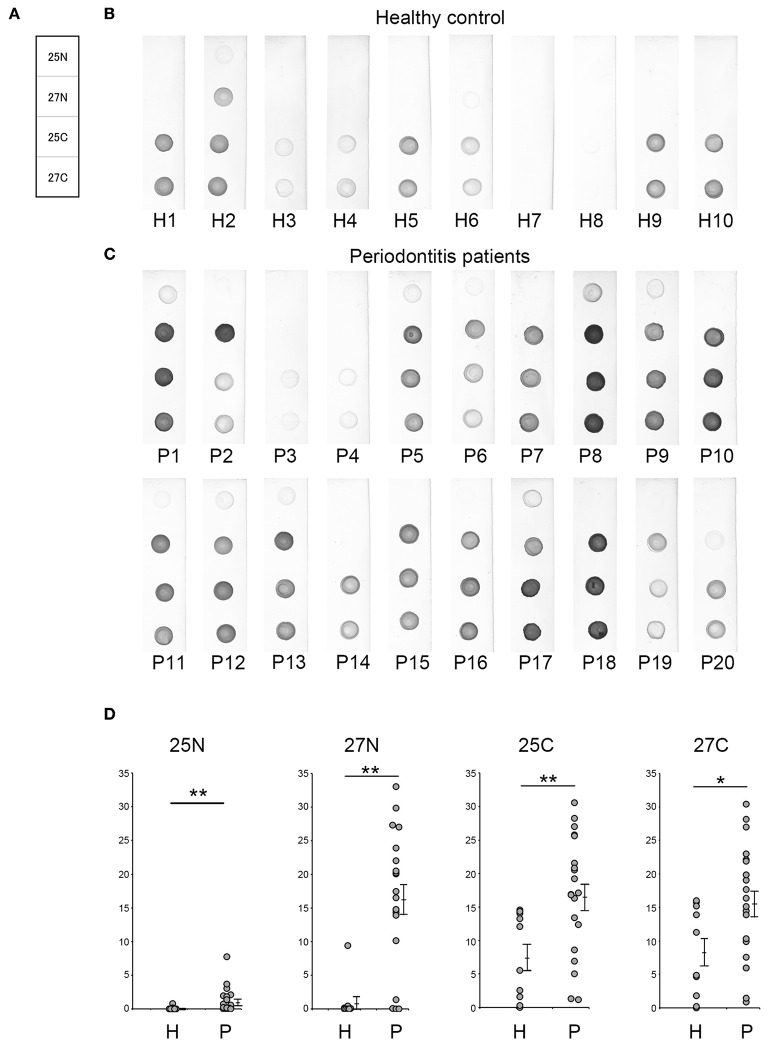
Serum IgG responses to the recombinant antigens as determined by dot blot analysis. **(A)** The panel shows a scheme of the proteins spotted. Representative results of the sera used from healthy individuals (**B**: H1–H10) and patients with periodontitis (**C**: P1–P20) are shown. Approximately 50 ng of each protein was spotted on the membrane, and sera were used at a dilution of 1:2,500. **(D)** Spot intensities were normalized relative to the background signals and represented as relative intensities. Mean values and standard errors are shown on the right. **P < 0.01, *P < 0.05, N, N-terminal half; C, C-terminal half; H, Healthy control; P, Patients with periodontitis.

**Figure 7:**
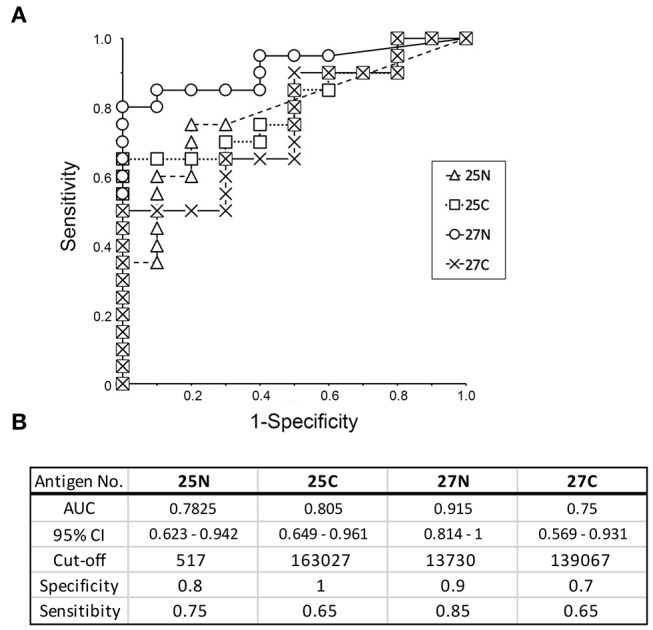
Receiver operative characteristic (ROC) curve analysis to evaluate the diagnostic capacity of individual recombinant antigens (25N, 25C, 27N, and 27C) to distinguish between periodontitis patients and healthy individuals. **(A)** ROC curves were generated as described by Ekuseru-Toukei 2010 (Social Survey Research Information Co., Ltd.) using the spot intensities from the dot blot analysis (Figure 6). **(B)** The values of AUC with 95% confidence interval (CI) and sensitivity/specificity with optimal cut-offs are presented in the lower panel. N, N-terminal half; C, C-terminal half.

In order to provide recombinant proteins stably in high yield using wheat germ expression system, we synthesized the half size of Kgp (25N and 25C) and RgpA (27N and 27C). The N-terminal fragments of gingipains including the catalytic domains encompassed amino acid residues from 1 to 856 and 1 to 855 of the Kgp and RgpA nascent polypeptide chains, respectively. On the other hand, C-terminal fragments started from 862 to 1718 (Kgp) and from 862 to 1703 (RgpA) residues, respectively and contained hemagglutinin/adhesin region. Using the half size of recombinant Kgp (25N and 25C) and RgpA (27N and 27C), dot blot analysis and ROC/AUC analysis were performed the same way as with the full-length of Kgp and RgpA. As shown in Figure 6, the half size of recombinants, except for No. 25N, showed almost the same properties on immunogenicity and serum IgG antibody levels as the full length of No. 25A and 27A, respectively. Interestingly, ROC/AUC analysis of antigen No. 27N showed outstanding discriminatory power (AUC = 0.915) on diagnostic ability to separate periodontitis patients from the healthy control group (Figure 7), which was significantly increased compared to antigen No. 27A described in Figure 5.

## Discussion

Multiple lines of evidence, including clinical studies, have shown that the progression of periodontal disease increases the risk of systemic diseases such as diabetes mellitus and Alzheimer's disease (2–7). Thus, there has been increasing amount of effort from medical specialists to control the occurrence of systemic diseases based on the periodontal status of individuals. However, an ideal biochemical marker for detecting the presence of periodontal disease has not yet been established. The components in gingival crevicular fluid (GCF) and saliva also have been investigated as potential biomarkers at predicting disease progression (33–36). GCF provides site specific information that represents disease related proteins (33–35). Saliva is considered as more of an overall assessment of the periodontal disease status (36). Many of the molecules reported as potential biomarkers in GCF or saliva were the products of host cells exuded by the destruction of periodontal tissue and the products derived from the subgingival microbial plaque (33, 36). Therefore, those molecules may reflect the disease pathophysiology. Meanwhile, periodontitis is an inflammatory response against periodontal pathogens, that causes alveolar bone loss. In this aspect, we have previously reported that serum IgG antibody level against *P. gingivalis* by ELISA showed the level of periodontal inflammation and is an appropriate marker for screening periodontitis patients (14). Although blood collection is invasive compared to taking a GCF or a saliva sample, it is minorly invasive and commonly performed in medical facilities. Furthermore, 50 μL of blood from fingertip is also good enough for this IgG test (14, 37). However, testing the serum IgG level for periodontal pathogens is not commonly performed during a clinical examination except for research purposes. This is one of the reasons behind the fact that crude whole protein extracts from *P. gingivalis* have been used as antigen sources for testing the IgG level by ELISA. This causes the test results to vary among institutes. In order to dissolve this problem, we considered the use of single or a combination of a small number of purified antigens. Although several antigens in *P. gingivalis* have been reported to have high immunogenicity, it is not clear which *P. gingivalis* antigens affect serum IgG levels in periodontitis patients.

In this study we utilized immunoaffinity columns immobilized with IgG antibodies from periodontitis patients and identified 29 antigens by Liquid chromatography-tandem mass spectrometry. In addition, we succeeded in synthesizing 29 recombinant proteins including stabilized gingipain (Kgp and RgpA) by point mutation. We then examined serum IgG levels against each of the 16 antigens, which exhibited strong immunogenicity in Figure 2B, using serum from 10 healthy individuals and from 20 periodontitis patients (comprising 10 patients each with mild alveolar bone loss or severe alveolar bone loss). Out of the 16 candidates, the serum IgG levels in periodontitis patients against Omp28 (antigen number 20), Kgp (antigen number 25), and RgpA (antigen number 27) were significantly higher as compared to healthy individuals (Figure 4); serum IgG levels did not correlate with the severity of alveolar bone loss (data not shown). Consistent with previous studies, serum IgG levels did not reflect the extent of tissue damage (38).

Omp28 is a 28 kDa outer membrane protein found in *P. gingivalis*. This protein is a surface adhesion/receptor protein (39) and is expressed in a variety of *P. gingivalis* strains. Based on extensive database searches, no protein exhibited high homology with Omp28 in other bacterial strains (39). Therefore, Omp28 has been a potential candidate for the unique diagnostic for periodontitis. Although serum IgG levels against Omp28 were significantly higher than those in healthy subjects (*p* = 0.009), half of the serum samples from patients (10 out of 20 periodontitis patients) did not recognize Omp28, indicating high false negative rates in this study. Moreover, Kgp and RgpA are well-known cysteine proteases in *P. gingivalis* and are the major virulence factors responsible for hindering the innate host defense mechanisms (40, 41). High serum IgG levels have been reported against gingipains in periodontitis patients (42, 43). In this study, we demonstrated that serum IgG levels against gingipains were the highest compared to a variety of *P. gingivalis* antigens that were synthesized using the same translation machinery. ROC curves were drawn to determine the cut-off based on the serum IgG level values against the recombinant antigens. The AUC was derived from the ROC curve that determined the ability of the antigen to discriminate between periodontitis patients and healthy subjects and diagnose disease. Kgp (AUC value of 0.78) and RgpA (AUC value of 0.815) showed moderate discrimination and were the highest compared to the AUC values of other antigens (Supplementary Figure 3). Using an optimal cut-off, the specificity of Kgp and RgpA was satisfactory, whereas the sensitivity was low (Figure 5).

Gingipains are large proteins (180 kDa) encoded by *kgp* or *rgpA* genes that comprise a signal peptide, N-terminal pro-fragment, Lys- or Arg-specific catalytic domain, and C-terminal multi-domain hemagglutinin/adhesin (HA1-HA4) region. It has been observed that serum IgG antibodies in periodontitis patients are primarily reactive to the hemagglutinin/adhesin region of Kgp and RgpA (44, 45). The adhesion domain 1 (HA1) and adhesion domain 4 (HA4) of RgpA are significantly recognized by the serum IgG antibodies in periodontitis patients as compared to those in healthy subjects (46). The homology between the hemagglutinin/adhesin regions of the *kgp* and *rgpA* genes is quite high (65–100%), while the homology between the catalytic domains is low (32%). Moreover, the shorter templates stably synthesized recombinant proteins in high yields using the wheat germ expression system. The N-terminus (antigen 25N and 27N) mainly comprised the catalytic domain and the C-terminus (antigen 25C and 27C) mainly comprised the hemagglutinin/adhesin region. We determined the immunogenicity and induction of serum IgG levels as the full length Kgp and RgpA. As expected, the C-terminus of Kgp (25C) exhibited similar phenotypes as that of full length Kgp. Our data of Kgp was in accordance with previous studies that showed that the C-terminal hemagglutinin/adhesin region primarily induced serum IgG antibodies in periodontitis patients (36). Interestingly, the N-terminal catalytic domain of RgpA (27N) exhibited a higher value of AUC (0.915) than the C-terminus (AUC = 0.75) and other variants of Kgp (antigen 25N and 25C). Previous studies have demonstrated the reactivity of the serum IgG antibodies from periodontitis patients to the hemagglutinin/adhesin regions of Kgp and RgpA (44–46). Our results for RgpA did not contradict those from previous reports. These studies also showed the induction of serum IgG reactivity with the catalytic domain, although the quantification of the antibody level or serum IgG reactivity against each domain of RgpA and Kgp has been limited (44–46). In this study, the majority of the serum samples from periodontitis patients strongly reacted to both antigens (27N and 27C) to the same extent. Healthy subject sera exhibited a distinct reactivity with 27N and 27C. The reactivity of sera from healthy subjects against 27N was remarkably weaker compared to the reactivity against 27C, suggesting that antigen 27N imparts higher specificity. As a result, 27N was associated with a higher AUC (0.915) showing outstanding discrimination in screening periodontitis patients.

Taken together, we have demonstrated that serum IgG levels in periodontitis patients were the highest against recombinant Arg-gingipain (RgpA) among the antigens expressed in *P. gingivalis*. Moreover, the N-terminus of recombinant RgpA was the most appropriate antigen for screening periodontitis patients. Using this antigen (N-terminus of RgpA) could potentially lead to the development of an accurate and rapid diagnostic kit for periodontitis. Given that antibody levels did not correlate with the degree of bone loss in this study, clinical examination for the severity of periodontal disease may require the combination of several biomarkers. In contrast, at least, it is possible to identify *P. gingivalis* infection for the diagnosis of periodontitis-associated systemic diseases. Finally, further experiments need to be performed with a larger sample study to determine if this antigen may be a biomarker for periodontitis and to understand the link between periodontitis and systemic diseases.

## Data Availability Statement

All datasets generated for this study are included in the article/Supplementary Material.

## Ethics Statement

The studies involving human participants were reviewed and approved by Ethical Committee of Okayama University Graduate School of Medicine, Dentistry, and Pharmaceutical Sciences (approval no. 624). The patients/participants provided their written informed consent to participate in this study.

## Author Contributions

ST and TE contributed to conception and design of the present study. KH contributed to analysis and interpretation of data and wrote the initial draft of the manuscript. TY-T contributed to data collection and interpretation. ST and HM critically reviewed the manuscript. All authors approved the final version of the manuscript and agree to be accountable for all aspects of the work in ensuring that questions related to the accuracy or integrity of any part of the work are appropriately investigated and resolved.

## Conflict of Interest

The authors declare that this study received funding from Sunstar Inc. The funder had the following involvement with the study: TE was employee of Sunstar Inc. which owns patent rights to test kit using the recombinant antigens in this study. He contributed to conception and design of this study and was partially involved in data collection. However, the funder was not involved in the analysis and interpretation of data and the writing of this article, nor did they decide to submit it for publication. The remaining authors declare that the research was conducted in the absence of any commercial or financial relationships that could be construed as a potential conflict of interest.
