# Supplementary material for: Identification and Modification of Porphyromonas gingivalis Cysteine Protease, Gingipain, Ideal for Screening Periodontitis
Source: Front Immunol. 2020 Jun 5;11:1017. doi: 10.3389/fimmu.2020.01017 (PMC7290125; doi:10.3389/fimmu.2020.01017)
Supplement: Supplementary file 1 [file Data_Sheet_1.DOCX]

# Supplementary Figures and Tables

## Supplementary Tables

| **Supplementary Table 1.** Primer sets for antigen genes. All the primers contained the respective restriction sites and attB1/B2 sequences for the subsequent cloning steps. The attB1 sequence (5’-GGGGACAAGTTTGTACAAAAAAGCAGGCT-3’) was added to the 5’ ends of each forward primer and the attB2 sequence (5’-GGGGACCACTTTGTACAAGAAAGCTGGGT-3’) was added to the 5’ ends of each reverse primer. | | | |
| --- | --- | --- | --- |
| **Antigen No.** | **Restriction sites (Forward/ Reverse)** | **Forward (5'-3')** | **Reverse (5'-3')** |
| 1 | BamHI/ NotI | ggatccAAGAAAAAGAATTTTTTGCTTCTTGGC | gcggccgcTTACCCGATATGGATCTTTTCCGT |
| 2 | BamHI/ NotI | ggatccCTTACGAAACTAAAAACACTGCTACTT | gcggccgcCTACAGATCCACGTCCTGCTCG |
| 3 | XhoI/ NotI | ctcgagATGAAACGATATACAATAATTCTTGC | gcggccgcTTAGAAACGAGCCCCGGAATCTCC |
| 4 | XhoI/ SpeI | ctcgagAAAAGAAAACCGCTATTCTCAGCCC | actagtTTACGGCATCGCGGTTTTGATCG |
| 5 | BamHI/ NotI | ggatccAGGATCAAGCCCTCTCTGAAAACGATG | gcggccgcTCAGAAGCGATACGACATTGTCATTG |
| 6 | BamHI/ NotI | ggatccAAAGTAAAACATCTATTAGCTGCAT | gcggccgcTCAGAATGCTACCTGCACCTGTGTTA |
| 7 | SmaI/ SpeI | cccgggATGTCGTTACCGCAGCACAATCG | actagtTTAGAATTCGATATCATAGTTATG |
| 8 | BamHI/ NotI | ggatccAAAAGATTATTACTCTCTGCTGCTAT | gcggccgcTCAAGGAACTAAGACTTTAAGGAAAT |
| 9 | BamHI/ NotI | ggatccAAAAAACTGATTTTAGCGACTT | gcggccgcTTAGCGAGCAGAGGTGGCTTTCATA |
| 10 | BamHI/ NotI | ggatccAAGACCCAAGAAATTATGACAATGC | gcggccgcTTAGCAAACGCCCTGAGCTACCATA |
| 11 | BamHI/ NotI | ggatccAAGAAGCACAATTTCACCGCAGGACC | gcggccgcTTAGTGCTTAGCTTCGAATTCCTTCAT |
| 12 | XhoI/ SpeI | ctcgagAAAAAGTTTTTCTTCGCGCTACTATC | actagtTTAGAAGTTGACCATAACCTTACCCAT |
| 13 | BamHI/ NotI | ggatccAAAAAAACAAAGTTTTTCTTGTTGGG | gcggccgcTTACCAAGTAGCAGCCTGATTAACAACAACCCA |
| 14 | BamHI/ NotI | ggatccAAAAAATTGCTATATATACTGTTGC | gcggccgcTTACGGATTCGCCACCGTTATCGTCTG |
| 15 | BamHI/ NotI | ggatccAAAAAAATCATTTTCTCCGCACTCT | gcggccgcTTATTTAACGGGGTATGTATAAGC |
| 16 | BamHI/ NotI | ggatccACAAACGATATCTTGCAGCGTCTTGCG | gcggccgcTTAGATAGTCTGAGTTTTCTCTTTGA |
| 17 | BamHI/ NotI | ggatccACGAAAGTAGGTATTAACGGCTTTG | gcggccgcTTATGCGTTTACCTTAGCCATGTAG |
| 18 | BamHI/ NotI | ggatccAAAATAAGCGAAAACGTAACTAAAGC | gcggccgcTTATCTCCGGGCGAGCTGATGATCGATG |
| 19 | SmaI/ SpeI | cccgggAAAAAAGACAGCGTAATTTTCGAT | actagtTTACCAAGCAAAGAGAGGATAATCGGC |
| 20 | SmaI/ SpeI | cccgggAAAAAGCTATTTCTCTCGCTCACG | actagtTTATTTGCCATCGGATTGCGGATTGA |
| 21 | SmaI/ SpeI | cccgggAACAAATTTTACAAATCACTTTTGC | actagtCTACTGCTTCACGATCTTTTGGCTCACA |
| 22 | SmaI/ SpeI | cccgggAAAAAAACGCTCGTAATAGTCG | actagtTCATTCTCCTTTGATAAAGCGG |
| 23 | XhoI/ SpeI | ctcgagAAAAAGATTCTTGAAGTAACGGGT | actagtTTACTTGGTAGCGTAGGCAGACAGC |
| 24 | XhoI/ SpeI | ctcgagAAAGCTAAATCTTTATTATTAGCACT | actagtTTATTCCGCTGCAGTCATTACTACAA |
| 25 | XhoI/ SpeI | ctcgagAGGAAATTATTATTGCTGATCGCGGC | actagtTTACTTGATAGCGAGTTTCTCTACG |
| 26 | XhoI/ NotI | ctcgagAAAAGAATGACGCTATTCTTCCTTTG | gcggccgcTTAGAAAGAAATCTGAATACC |
| 27 | XhoI/ SpeI | ctcgagTTGAACAAGTTTGTTTCGATTGCT | actagtTTACTTTACAGCGAGTTTCTCTACG |
| 28 | XhoI/ SpeI | ctcgagAAGGTAAAGTACTTAATGCTCACA | actagtTTACTTGGAGCGAACGATTACAACACG |
| 29 | XhoI/ SpeI | ctcgagAAAAAAATAATTTATTGGGTTGCGACAGTTT | actagtTTATATCGGCCAGTTCTTTATTAACTGCGGATT |
| 25N | XhoI/ SpeI | ctcgagAGGAAATTATTATTGCTGATCG | actagtTTACTTCTTGCCTGCTTCGAATGTG |
| 25C | XhoI/ SpeI | ctcgagTACACCTTCACGATGCGTCG | actagtTTACTTGATAGCGAGTTTCTC |
| 27N | XhoI/ SpeI | ctcgagAAAAACTTGAACAAGTTTGT | actagtTTAACCTTCGCTTATAGTCAATTC |
| 27C | XhoI/ SpeI | ctcgagGGTGGAAGCGATTACACCTA | actagtTTACTTTACAGCGAGTTTCTC |

## Supplementary Figures

**
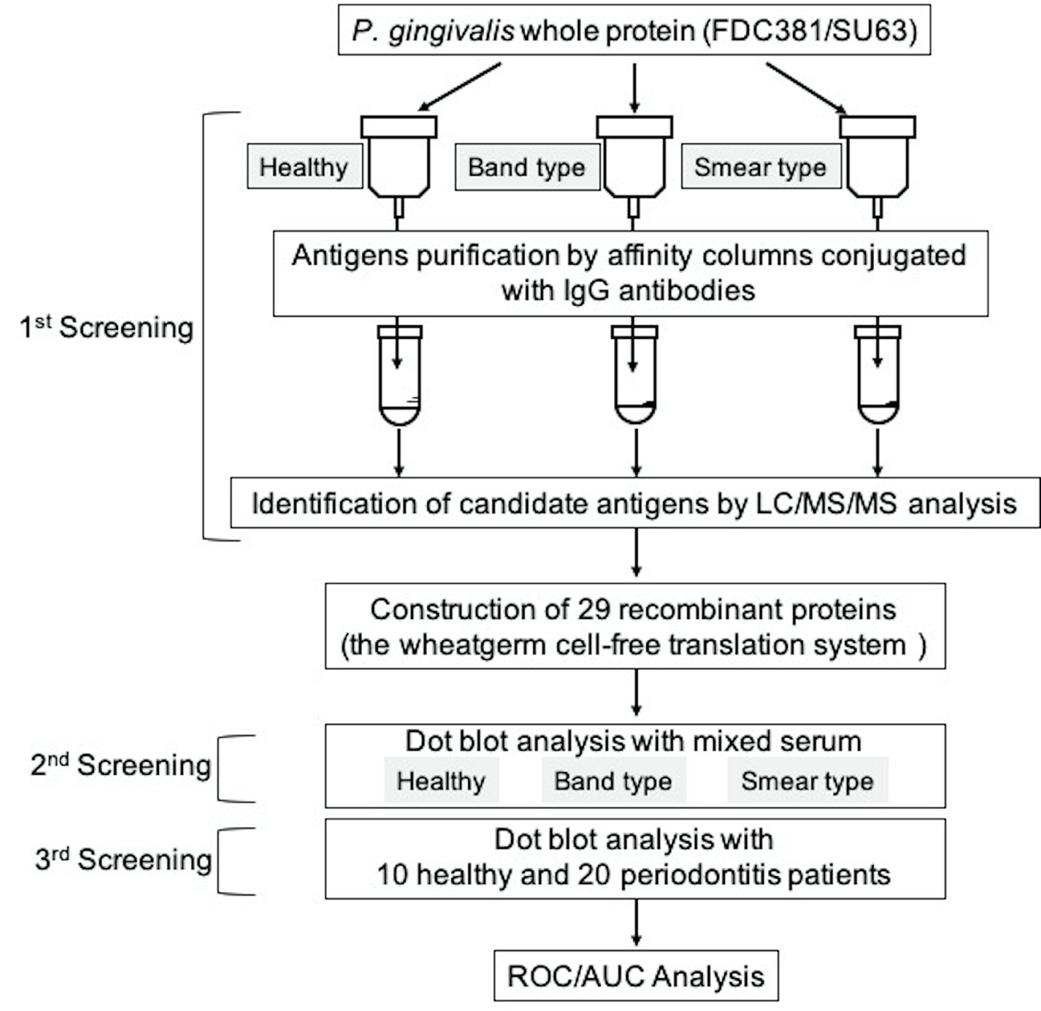
**

**Supplementary Figure 1.** Chart showing the schematic procedures followed in the study. This study comprised 3 screening steps to identify the antigens specifically recognized by periodontal patient serum IgG antibodies compared to healthy subjects that could be used as novel antigens for diagnosing periodontitis. We used two different types of serum from periodontitis patients that exhibited two recognition patterns (clear bands and smears) in the 1^st^ and 2^nd^ levels of screening against the whole protein extracts of *Porphyromonas gingivalis*. The two different immunoreaction patterns are shown in supplementary Figure 2. The 3^rd^ screening step utilized 20 periodontal patients including 10 with mild (18.6 ± 7.2) or severe alveolar bone loss (51.0 ± 21.5) measured by the method of Schei.

**
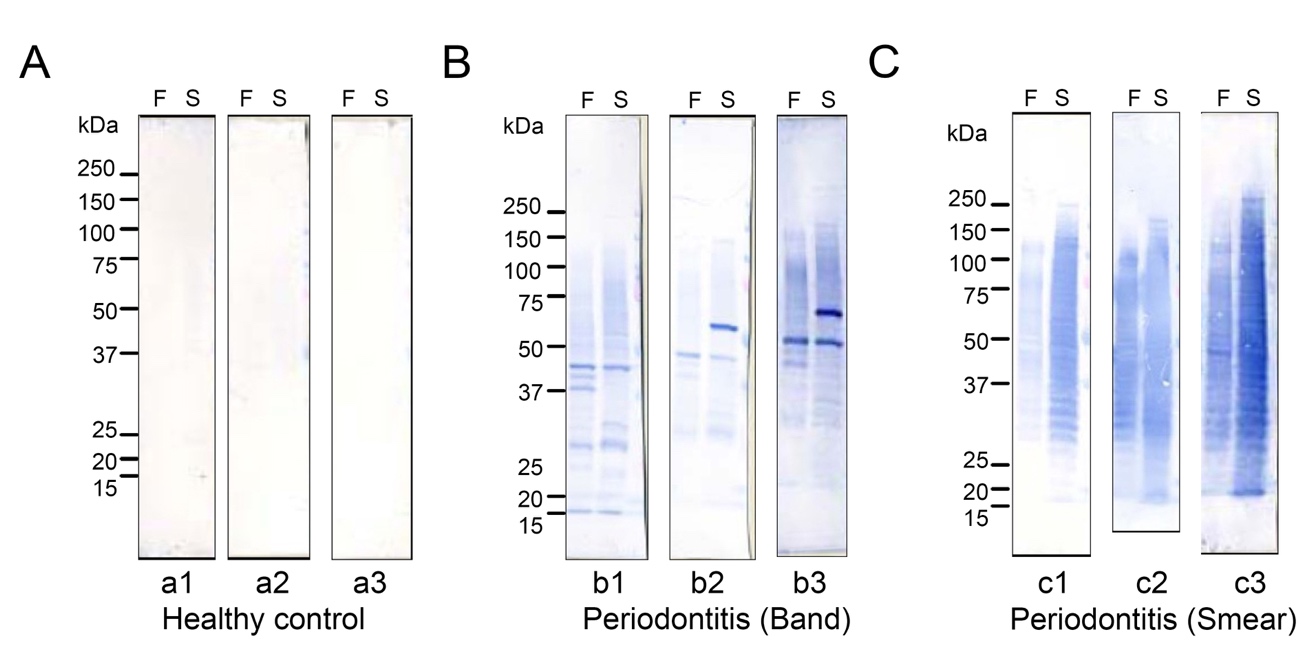
**

**Supplementary Figure 2.** Serum IgG responses to *P. gingivalis* were examined by western blot analysis. Human serum from patients with periodontitis exhibited two different immunoreaction patterns against *P. gingivalis*. Representative results of serum from healthy controls (A), patients with periodontitis, which exhibited the clear band on the blot (B), and the smear on the blot (C) are shown. F: *P. gingivalis* FDC381, S: *P. gingivalis* SU63.

**
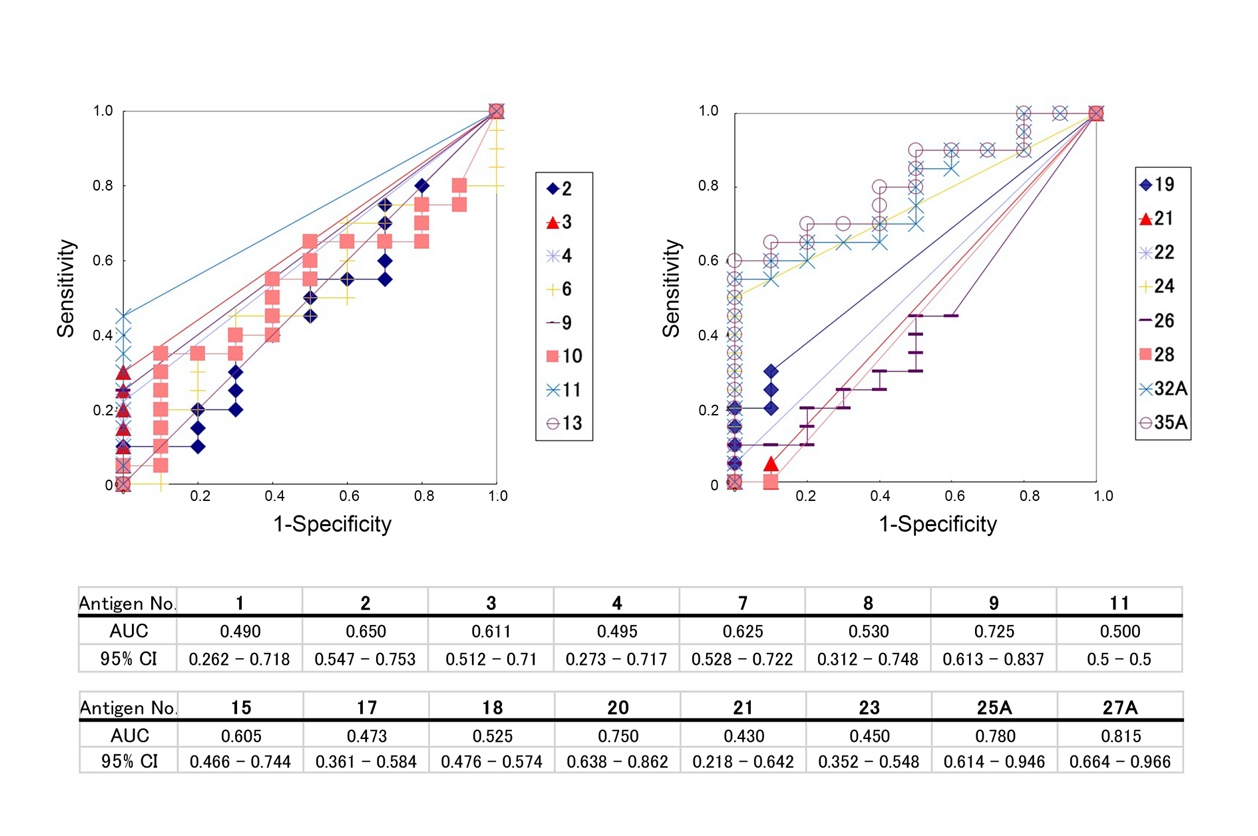
**

**Supplementary Figure 3.** Diagnostic ability for the individual recombinant antigens to separate periodontitis patients from healthy control was analyzed by Receiver operative characteristic (ROC) curve analysis. ROC curve (A) and Area under the ROC curve (AUC) with 95% confidence interval (CI) (B) were generated by Ekuseru-Toukei 2010 (Social Survey Research Information Co., Ltd.) using the spot intensity on the dot blot analysis (Figure 6).
